# Barriers and facilitators of medication adherence in the management of Diabetes among the Brazilian Amazon population

**DOI:** 10.15649/cuidarte.5207

**Published:** 2026-04-21

**Authors:** Raquel Coelho de Andrade, Thalyta Mariany Rêgo Lopes Ueno, Tiago Assunção dos Santos Farias, Wagner Ferreira Monteiro, Hércules Lázaro Morais Campos, Elisa Brosina De Leon

**Affiliations:** 1 Faculdade de Educação Física e Fisioterapia, Universidade Federal do Amazonas, Manaus, Brazil. E-mail: raquelandrads13@gmail.com Universidade Federal do Amazonas Manaus Brazil raquelandrads13@gmail.com; 2 Programa de Pós-graduação em Enfermagem em Saúde Pública, Escola de Ciências da Saúde, Universidade do Estado do Amazonas, Manaus, Brazil. E-mail: tueno@uea.edu.br Universidade do Estado do Amazonas Manaus Brazil tueno@uea.edu.br; 3 Programa de Pós-graduação em Ciências do Movimento Humano, Faculdade de Educação Física e Fisioterapia, Universidade Federal do Amazonas, Manaus, Brazil. E-mail: tfarias92@gmail.com Universidade Federal do Amazonas Manaus Brazil tfarias92@gmail.com; 4 Programa de Pós-graduação em Enfermagem em Saúde Pública, Escola de Ciências da Saúde, Universidade do Estado do Amazonas, Manaus, Brazil. E-mail: wfmonteiro@uea.edu.br Universidade do Estado do Amazonas Manaus Brazil wfmonteiro@uea.edu.br; 5 Instituto de Saúde e Biotecnologia, Universidade Federal do Amazonas, Coari, Brazil; Programa de Pós-Graduação Interdisciplinar em Estudos Rurais, Universidade Federal dos Vales do Jequitinhonha e Mucuri, Diamantina, Minas Gerais, Brazil. E-mail: herculeslmc@hotmail.com Universidade Federal do Amazonas Minas Gerais Brazil herculeslmc@hotmail.com; 6 Programa de Pós-graduação em Ciências do Movimento Humano, Faculdade de Educação Física e Fisioterapia, Universidade Federal do Amazonas, Manaus, Brazil. E-mail: elisadleon@ufam.edu.br Universidade Federal do Amazonas Manaus Brazil elisadleon@ufam.edu.br

**Keywords:** Healthy Lifestyle, Vulnerable Population, Noncommunicable Diseases, Primary Health Care, Healthcare Disparities, Estilo de Vida Saludable, Población Vulnerable, Enfermedades no Transmisibles, Atención Primaria de Salud, Disparidades en la Atención de Salud, Estilo de Vida Saudável, População Vulnerável, Doenças não Transmissíveis, Atenção Primária à Saúde, Disparidades na Assistên à Saúde

## Abstract

**Introduction:**

Type 2 diabetes Mellitus (T2DM) is a prevalent chronic condition with global impact. Medication adherence is essential for disease control, but it remains a challenge in socially vulnerable contexts such as the Brazilian Amazon.

**Objective:**

To identify barriers and facilitators of medication adherence in the management of T2DM in the Amazon population.

**Materials and Methods:**

A qualitative, descriptive study was conducted in Iranduba (Amazonas) involving individuals with T2DM and community health workers (CHWs). The World Café method and semi-structured interviews were used. Data were analyzed with the support of ATLAS.ti software, using Thematic Network Analysis.

**Results:**

A total of 64 participants were included (47 individuals with T2DM and 17 CHWs). Reported barriers included difficulty remembering medication schedules, adverse effects, limited financial resources, lack of medication availability in primary care, and insufficient professional support. Facilitators included self-management strategies, commitment to treatment, purchasing power, family support, and CHWs' actions.

**Discussion:**

Medication adherence in Diabetes is shaped by barriers such as forgetfulness, adverse effects, and limited access to medications, while family support, professional guidance, and personal strategies facilitate treatment. These factors interact and affect self-care.

**Conclusion:**

Medication adherence among individuals with T2DM in the Amazon is shaped by intrapersonal, interpersonal, and environmental factors. Culturally adapted strategies, greater professional support, and public policies ensuring medication availability are essential to improving care and preventing complications.

## Resumen

**Introducción:**

La Diabetes Mellitus Tipo 2 (DM2) es una condición crónica prevalente y de impacto mundial. La adherencia a la medicación es fundamental para el control de la enfermedad, pero sigue siendo un desafío en contextos de vulnerabilidad social, como en la Amazonia Brasileña.

**Objetivo:**

Identificar barreras y facilitadores de la adherencia a la medicación en el manejo de la DM2 en la población amazónica.

**Materiales y métodos:**

Estudio cualitativo, descriptivo, realizado en Iranduba (Amazonas), con personas con DM2 y agentes comunitarios de salud (ACS). Se emplearon la técnica del World Café y entrevistas semiestructuradas. El análisis de datos se realizó en el software ATLAS.ti, empleando Análisis de Redes Temáticas.

**Resultados:**

Participaron 64 individuos (47 personas con DM2 y 17 ACS). Las barreras identificadas incluyeron dificultad para recordar los horarios de medicación, efectos adversos, falta de recursos financieros, escasez de medicamentos en la atención primaria y apoyo profesional insuficiente. Como facilitadores se destacaron estrategias de organización, compromiso con el tratamiento, poder adquisitivo, apoyo familiar y el acompañamiento de los ACS.

**Discusión:**

La adherencia medicamentosa en la Diabetes está influenciada por barreras como el olvido, los efectos adversos y el acceso limitado, mientras que el apoyo familiar, la orientación profesional y las estrategias personales favorecen el tratamiento.

**Conclusiones:**

La adherencia al tratamiento medicamentoso en personas con DM2 en la Amazonia está influenciada por factores intrapersonales, interpersonales y ambientales. Estrategias culturalmente adaptadas, mayor apoyo profesional y políticas públicas que garanticen la disponibilidad de medicamentos son esenciales para mejorar la atención y prevenir complicaciones.

## Resumo

**Introdução:**

O diabetes mellitus tipo 2 (DM2) é uma condição crônica prevalente com impacto global. A adesão à medicação é essencial para o controle da doença, mas permanece um desafio em contextos socialmente vulneráveis, como a Amazônia brasileira.

**Objetivo:**

Identificar as barreiras e os facilitadores da adesão à medicação no manejo do DM2 na população da Amazônia.

**Materiais e métodos:**

Foi realizado um estudo qualitativo e descritivo em Iranduba (Amazonas) com indivíduos com DM2 e Agentes Comunitários de Saúde (ACS). A coleta de dados incluiu o método World Café e entrevistas semiestruturadas. A Análise Temática em Rede foi realizada utilizando o software ATLAS.ti.

**Resultados:**

Foram incluídos 64 participantes (47 indivíduos com DM2 e 17 ACS). As barreiras relatadas incluíram dificuldade em lembrar os horários de medicação, efeitos adversos, recursos financeiros limitados, falta de medicamentos na atenção primária e apoio profissional insuficiente. Os facilitadores incluíram estratégias de automanejo, comprometimento com o tratamento, poder aquisitivo, apoio familiar e assistência dos ACS.

**Discussão:**

A adesão à medicação no diabetes é influenciada por barreiras como esquecimento, efeitos adversos e acesso limitado, enquanto o apoio familiar, a orientação profissional e as estratégias pessoais facilitam o tratamento.

**Conclusões:**

A adesão à medicação entre indivíduos com diabetes tipo 2 na Amazônia é influenciada por fatores intrapessoais, interpessoais e ambientais. Estratégias adaptadas culturalmente, maior apoio profissional e políticas públicas que garantam a disponibilidade de medicamentos são essenciais para melhorar o cuidado e prevenir complicações.

## Introduction

Type 2 diabetes mellitus (T2DM) accounts for approximately 90 to 95% of all diabetes cases and represents a global public health problem^1^. The projected global estimate for 2045 is 700 million cases^2^. In Brazil, 23.2 million people are expected to be affected by the disease by the same year^3^. The state of Amazonas, in the Brazilian Amazon, currently has more than 185,000 people with T2DM^4^.

Population aging and deleterious lifestyle habits contribute to an increase in this chronic disease, causing long-term health complications^5^ and generating economic problems for both patients and their families, as well as for public health, which faces costs related to hospitalization, outpatient treatment, and medication^6^. T2DM management encompasses lifestyle modifications, including dietary changes, regular physical activity, and medication adherence^3^.

Adherence to therapy is a determining factor in maintaining metabolic control and ensuring treatment efficacy^7^. However, despite treatment being widely known, medication adherence remains a challenge, especially among socially vulnerable populations, such as those living in the Brazilian Amazon^8^. Low adherence or inadequate adherence to glucose-lowering agents is influenced by several factors, including sociodemographic conditions, disease perception, and the complexity of the prescribed therapy^9^-^13^.

Recognizing the specificities of each context, especially in vulnerable regions, is essential to improve the care strategies offered, considering the lack of human and financial resources and the difficulties in access^14^,^15^. In this sense, identifying barriers and facilitators to medication adherence in the Brazilian Amazon enables researchers and health professionals to intervene and map difficulties and opportunities for transforming health promotion in these settings, making it more efficient and effective.

Understanding the barriers and facilitators that influence individuals' ability to incorporate these changes into their daily lives is crucial, as strategies must be tailored to each person and their unique realities, which are a determining factor in the success of interventions. This study aims to identify the barriers and facilitators of medication adherence in the management of diabetes among populations in the Brazilian Amazon.

## Materials and Methods

This research is a qualitative, descriptive study part of a larger research project entitled “Intervention led by community health agents for the management of Type 2 Diabetes in the interior of the Amazon," led by Dr. Elisa Brosina de Leon. This research was conducted in accordance with the Consolidated Criteria for Reporting Qualitative Research (COREQ) checklist.

The study setting was a municipality in the northern region of Brazil, Iranduba, located 19.89 km from the capital of Amazonas. The municipality was selected as it represents a cultural and sociodemographic context comparable to that of other municipalities in Amazonas^16^. Iranduba has 9 Primary Healthcare (PHC) units within its territory and, like other municipalities, faces challenges due to its distance from the capital, the lack of medicines and supplies in the PHC units, and a shortage of professionals^15^.

To better understand the barriers and facilitators to medication adherence in the Amazonian context, the study included people diagnosed with T2DM and community health workers (CHWs) employed in PHC units. The study included people aged over 18 with a diagnosis of T2DM for at least six months; individuals with physical, intellectual, or communicative disabilities that interfered with understanding and participation were excluded, and CHWs working at the PHC units.

Data collection employed two qualitative techniques: the World Café method and semi-structured interviews. The World Café is a collaborative conversational method in which participants gather in groups of four or five around small tables, similar to those found in coffee shops. They discuss important issues and collaborate to build collective knowledge about specific problems^17^.

World Café meetings, referred to as 'Prosa e Café,' were scheduled at times compatible with the service hours of the PHC units and conducted following authorization from the Municipal Health Department. The host researcher engaged the CHWs via telephone and a WhatsApp group, followed by an online meeting with the CHWs to introduce the research team and provide the information needed to understand the dynamics.

The meetings include people with T2DM and CHWs. In the first meeting, the question asked was: 'What are the barriers to medication adherence among people with T2DM?' In the second meeting, the question asked was: 'What are the facilitators to medication adherence among people with T2DM?' Both questions were used during the World Café sessions, during which participants also completed a sociodemographic questionnaire. For the semi-structured interviews, an interview script was developed for this study, consisting of two parts: the first to gather sociodemographic data, and the second to address barriers and facilitators to adherence to T2DM medication.

Four World Café sessions were conducted. On the first day, in the morning, individuals with T2DM discussed barriers to medication adherence, while CHWs participated in the afternoon. On the second day, both groups explored facilitators of medication adherence. Finally, the findings were shared and consolidated in a plenary session.

Semi-structured interviews were conducted during home visits with individuals with T2DM, based on a list provided by the CHWs. Due to the need to delve deeper into the data collected during the World Café sessions with people with T2DM, additional semi-structured interviews were conducted. Individuals invited to the interviews did not participate in the World Café sessions and were selected from among people with T2DM who experienced difficulties adhering to T2DM treatment, as reported by the CHWs. The interviews were conducted by trained physiotherapy and nursing students. Participants were contacted by telephone to schedule visits, and the interviews were recorded on the researchers’ tablets using voice recorders. At the end of each day, the recordings were uploaded to Google Drive for secure storage.

The Qualitative Data Analysis & Research Software (ATLAS.ti) was used to manage the data, following the principles of Thematic Network Analysis. Thematic Network analysis is a sensitive tool for systematizing and presenting qualitative data analyses. It shares with thematic analysis the identification of themes at different levels, the exploration of understandings of issues or conceptual meanings, and the provision of additional resources for structuring and representing these themes^18^.

The software enabled the creation of a hermeneutic unit for data processing. First, after the interviews were transcribed and reviewed, a provisional set of codes was developed deductively. The research team then reviewed and discussed the results to interpret the preliminary data. A second round of coding was conducted to look for patterns in the initial coding and identify themes, resulting in the Barriers and Facilitators Thematic Network for medication adherence. The Ecological Model was used to guide data collection, analysis, and message development^19^.

To preserve the confidentiality of the study participants, the letter “A” was used to identify CHWs and the letters “US” to identify the participants, followed by sequential numbers, representing the chronological order of the interviews. The complete dataset is freely available in Mendeley Data^20^.

## Results

**Figure 1:**
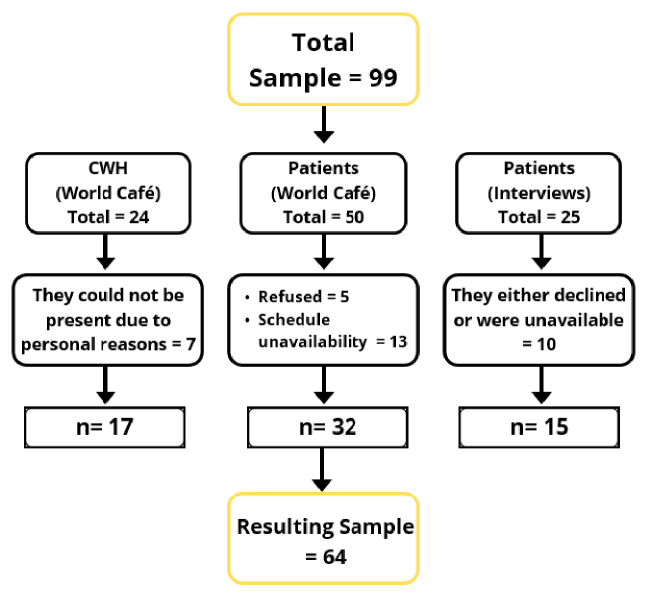
Flowchart of study participants, Iranduba, Amazonas, 2024.

Of the 50 individuals with T2DM invited by the CHWs, 32 participated in the group activities. In total, 49 participants were included: 32 individuals with T2DM and 17 CHWs Figure 1. For the interviews, the CHWs provided a list of 25 individuals with T2DM; however, 10 were either unavailable or declined to participate, resulting in 15 completed semi-structured interviews Figure 1.

**Figure 2:**
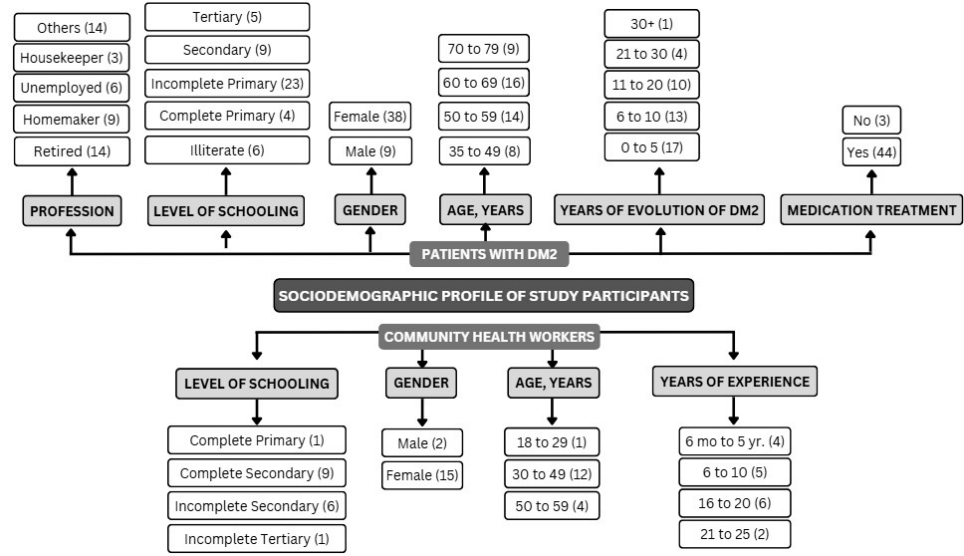
Sociodemographic profile of study participants, Iranduba, Amazonas, 2024.

Figure 2 presents the sociodemographic profile of the study participants. Among individuals with T2DM (n = 47), most were female (n = 38) and aged between 60 and 69 years (n = 16). Regarding educational attainment, the majority had incomplete primary education (n = 23), followed by secondary education (n = 9), while six participants were illiterate. The most common occupational statuses were retirees (n = 14) and homemakers (n = 9). Regarding disease duration, 17 participants had been living with T2DM for up to five years, and 13 had been living with the condition for between six and ten years. Almost all participants were receiving pharmacological treatment (n = 44).

Among CHWs (n = 17), most were female (n = 15) and aged 30 to 49 years (n = 12). Nine had completed secondary education, while six had incomplete secondary education. Regarding work experience, the largest group had 21 to 25 years of service (n = 2), followed by 16 to 20 years (n = 6).

**Figure 3:**
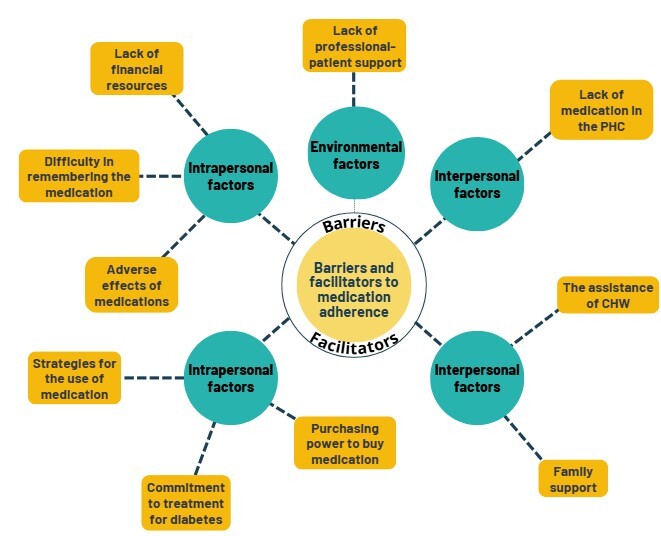
Thematic network of barriers and facilitators to medication adherence.

Figure 3 illustrates the barriers and facilitators to medication adherence identified in the study. Barriers were grouped into intrapersonal, environmental, and interpersonal factors.

Intrapersonal barriers included difficulty remembering to take medication and adverse effects. Adverse drug effects, as an intrapersonal factor, were present in the accounts of both participant groups and across the different data collection techniques. Participants reported that the adverse reactions to diabetes medications constitute a barrier to maintaining pharmacological treatment.

*US6: "Metformin gave me really bad diarrhea at first, a lot! Then I saw a doctor, and he thought about stopping the medication. He said it was a normal symptom. It would be normal until my stomach got used to it. And then I did get used to it... Now, this year, the diarrhea has started to ease up."*

Another barrier, classified as an intrapersonal factor, and identified in conversations with people with T2DM and CHWs, was struggling to remember taking medication regularly and at the prescribed times, according to the following statements:

*US3: "Sometimes I remember... until it’s time to take the medication. I remember, then I take it. But that’s how is it. I always forget to take the medication at the right time."*

The lack of financial resources to purchase medications was identified as a barrier in the accounts of both participant groups and was classified as an intrapersonal factor. It was noted that low income and high medication costs affect adherence.

*US13: "Yes, because when I’m at home, I have nothing to buy, right? Nowadays, there’s no money for anything, right? So sometimes I go a day or two without taking the medicine [...] Yeah, because sometimes there isn’t any at home for me to take, right? Then sometimes I don‘t have any money, so I go and tell my daughter, and she buys it, right? Then I start taking it, and it controls it again."*

Regarding interpersonal factors, the lack of support from health professionals for individuals with T2DM was highlighted as a barrier in the study. It was reported that the lack of follow-up, assistance, and necessary guidance on prescribed medications constitutes an obstacle to appropriate adherence to medication.

*US8: "...Other doctors come here, but they barely look at you, right? You say, ‘I’m in pain here, I’m in pain there,’ and they don’t even look at the person or talk to them. They wait for you to finish speaking, and by the time you’re done, they already have a prescription in their hand; they don’t even show the slightest willingness to run a test and check how the person is."*

Regarding environmental factors, the lack of medication availability at the PHC units was a barrier mentioned by both people with T2DM and CHWs. The lack of medications in the public health network interrupts the treatment for those who cannot afford them.

*A8: "...Another lack we’ve identified, this barrier, is not having medications at the PHC unit..."*

*US9: "...Difficulty getting medication at the PHC unit. I look for medication at the PHC unit, but there isn’t medication. Difficulty getting medication at the PHC unit..."*

Facilitators were also categorized into intrapersonal, environmental, and interpersonal factors. Strategies to facilitate the correct use of medications were highlighted, including setting cell phone alarms, writing reminders, and keeping medications in a fixed and visible place at home; these strategies were emphasized to ensure consistent and appropriate medication use.

*US7: "I put the medicines into two boxes to make it easier to remember the times, and I leave them in a visible place."*

*A2: "It makes it easier for them to set alarms on their cell phones so they can take their medication on time."*

Concerning intrapersonal factors, another prominent theme was a commitment to treatment for diabetes control. This theme emerged both from the World Café participants and the semi-structured interviews. It was emphasized that following the guidelines, adhering to the prescriptions, and having healthy lifestyle habits facilitate medication adherence.

*US9: "I remember, I take one after lunch, when I finish lunch at 11 a.m. At 11:15 a.m., I take it [...] I already know the medications, I already know which ones to take. [...] And this other one is for diabetes... this one here... I already know everything. I follow the prescription. Thank God I have a good memory! So far, I remember everything!"*

Participants also identified their purchasing power to buy medications as an intrapersonal factor that facilitates medication adherence, given the frequent shortages of diabetes medication at the PHC units.

*US11: "But when there aren’t any, I have a bit more purchasing power, so I buy some and don’t stop taking it. I’m a retired civil servant, and I worked for many years."*

Regarding interpersonal factors, participants’ statements indicate that the assistance, care, follow-up, active listening, and guidance provided by health professionals, including CHWs, contribute to patients' adherence to correct medication use.

*A9: "Good guidance, well given, and good communication with them, they adhere, that’s it." *

*US7: "Then I went to the doctor at the PHC unit about fifteen or twenty days ago... maybe not even that long... I talked a lot to a doctor, and this doctor was a 'new' doctor, but I have to say, hats off to her. She was very attentive, one of the best doctors who has ever been here."*

Family support, as an interpersonal factor, stood out in the accounts of both participant groups. Assistance and support from family members were essential conditions for ensuring correct medication adherence, especially among older and/or illiterate participants.

*US3: "My daughter remembers the schedule; she keeps asking me if I’ve taken my medication. A9: "Family support. Why family support? Because not all patients can read, they may not know which medication to take. So, they depend, first of all, on their family being with them and encouraging them to take the medication. That’s very important."*

## Discussion

This study highlights various factors that facilitate and hinder adherence to pharmacological treatment among users of health services, especially in the context of diabetes. Barriers included difficulty remembering to take medication, adverse effects, lack of financial resources, insufficient professional support, and unavailability of medications in PHC units. Facilitators included commitment to treatment, medication-use strategies, purchasing power, family support, and assistance from CHWs. These factors are interconnected and interact, influencing individuals' daily lives and attitudes.

Intrapersonal factors include individuals' skills and behaviors that influence diabetes self-care. Interpersonal factors involve relationships with family members, friends, and health professionals, as well as the support provided through these interpersonal relationships, which can enhance self-care among individuals with diabetes^21^.

Environmental factors include work, school, and the community around the individual. Public policies play a crucial role in the health of people with diabetes by creating laws and investing in improvements to public services for the community^13^. Both participant groups identified an intrapersonal barrier: difficulty remembering to take medication. Similar results were reported in a study on medication adherence difficulties conducted among patients with diabetes in a municipality in the state of Bahia.

In that study, 65% of participants reported difficulty remembering to take their medication, and 45% reported forgetting the correct times to take it^22^.

One possible explanation is the complexity of the therapeutic regimen and the number of medications prescribed. The simpler the regimen, the greater the treatment adherence, facilitating understanding and, consequently, treatment success^23^. Patients with comorbidities tend to take multiple medications, which makes taking them at the correct times more challenging; as a result, adverse effects may be more frequent since older adults without guidance are more likely to use medications without a routine, thereby interfering with the drug’s effectiveness in the body^24^.

People with T2DM also highlighted adverse drug reactions as an intrapersonal barrier that affects the frequency of pharmacological treatment. The adverse effects of antidiabetic medications, such as nausea, gastrointestinal discomfort, and emotional disturbances, are important factors influencing treatment continuity. These adverse effects reduce patients' quality of life and decrease their motivation to maintain adherence to prescribed medications^25^.

In addition, adverse effects can lead to a perception of treatment ineffectiveness, increasing medication discontinuation. Pharmacological therapy, often involving multiple daily doses and restrictions, can further exacerbate this situation, making it difficult for people with T2DM to follow medical recommendations consistently^26^. Health professionals must provide ongoing support, adjust therapies, and educate patients on managing these effects, thereby promoting better adherence and more favorable outcomes^27^.

Furthermore, most patients in this study considered developing strategies for the correct and regular use of medication as an intrapersonal facilitator. Similar results were found in studies examining per-ceptions of medication adherence among people living with diabetes, in which they used "clues" and visual reminders to take their medication at the correct times^23^. These strategies reflect the patient's commitment to treatment and are also facilitators of adherence among the participants.

When individuals with T2DM show a high level of commitment to their health, they tend to follow medical advice more rigorously, adopt healthy lifestyle habits, and take their medications correctly^11^. These behaviors help ensure treatment effectiveness and disease control. Educational level can also significantly influence treatment adherence, as education is associated with a better understanding of medical instructions and the benefits of treatment^28^.

A high level of education is also associated with better access to health resources and stronger social support. According to participants’ reports, purchasing power is likewise a facilitator of medication adherence, especially during medication shortages at PHC units^29^. Studies on factors that impact adherence to pharmacological treatment among people with T2DM in public health institutions have shown that patients who have to use their own financial resources to purchase medication tend to value treatment more and adhere to medical recommendations^30^.

Regarding interpersonal factors, the assistance of health professionals was highlighted as a facilitator of improved medication adherence. When professionals adopt a holistic approach to patient care, patients feel welcomed and are better informed about the implications and consequences of adhering effectively to medication^31^.

The presence of CHWs in the routines of people with T2DM, providing ongoing guidance on medication dosage and administration times, promotes consistent adherence. A study on the role of CHWs in community health also found similar results, emphasizing the importance of effective communication between health professionals and patients to improve treatment adherence^32^. Health professionals' welcoming and attentive attitudes can also positively influence perceptions and adherence^33^.

Family support emerges as a facilitator for older adults with T2DM who have low levels of education and limited resources. Family involvement in treatment contributes to improved patient health outcomes. Despite being active agents in self-managing their health, patients need support to motivate and encourage them to continue treatment, thereby reducing the risk of errors in medication use^34^,^35^.

Concerning environmental factors, the low availability of medications at PHC units was highlighted as a barrier by both participant groups. Studies show that the lack of medications at PHC units in rural municipalities can be attributed to various interrelated factors, such as difficulties accessing rural areas, poor PHC infrastructure, and inefficient management, which may result in a lack of planning and inadequate replenishment, as well as budgetary constraints; these factors negatively impact the health of the municipality population dependent on these services^13^,^36^.

The primary limitation of this study is its geographic scope, as it was conducted exclusively in a single municipality within the state of Amazonas, Brazil. This restriction may limit the generalizability of the findings. However, previous studies conducted in the region have shown that different municipalities in Amazonas share similar demographic and social characteristics. Additionally, choosing a qualitative research method, particularly in a low-income population, presents challenges. Participants may be distrustful or reluctant to share personal information, especially if they are unsure how their data will be used or have had negative experiences with institutions. Furthermore, lower literacy levels and limited communication skills may hinder participants' understanding and engagement during interviews or focus groups, particularly when complex or unfamiliar topics are discussed.

## Conclusions

This research made it possible to understand the barriers and facilitators that influence adherence to pharmacological treatment among people with T2DM. Participants identified treatment complexity and medication adverse effects as barriers, especially among older adults. Continuous and clear su-pport from health professionals, along with strategies to simplify treatment regimens, can improve adherence. Facilitators were associated with the importance of family support, CHWs, and health professionals for effective self-care.

This study emphasizes the significance of public policies and health initiatives aimed at improving access to medications, enhancing professional support, and providing ongoing patient care. Additionally, it provides data to help devise strategies to mitigate and overcome the identified barriers.
